# Enhancing Stability of Pediatric Femoral Fractures Treated with Elastic Nail Using an External Fixator

**DOI:** 10.3390/jcm14041060

**Published:** 2025-02-07

**Authors:** Barak Rinat, Eytan Dujovny, Noam Bor, Nimrod Rozen, Avi Chezar, Guy Rubin

**Affiliations:** 1 Orthopedic Department, Emek Medical Center, Afula 1834111, Israel; eytan.dujovny1@gmail.com (E.D.); noambor@yahoo.com (N.B.); chezarmd@gmail.com (A.C.); guyrubinmd@gmail.com (G.R.); 2 Faculty of Medicine, Technion, Haifa 3525433, Israel

**Keywords:** diaphyseal femur fracture, elastic intramedullary nails, external fixation, pediatric

## Abstract

**Background**: Diaphyseal femoral fractures in children older than 5 years and before adolescence are usually treated surgically. The literature describes several surgical techniques; however, we present an additional minimally invasive technique that combines the use of elastic intramedullary nails and a uniplanar external fixator as an optional solution for managing more complex cases. **Method**: This was a retrospective review of four children aged 9–12 years who suffered from unstable diaphyseal femoral fractures and were admitted to our institution. **Results**: We treated four children between the years 2021 and 2023. All patients underwent closed reduction of their fractures and fixation with an elastic intramedullary nail and an external fixator. Full radiographic fracture healing with acceptable alignment was achieved in all patients. The minimum clinical follow-up was 1.5 years. No major complications were observed, and all patients achieved full clinical recovery as well as proper limb alignment and length. **Conclusions**: Fixation of complex diaphyseal femoral fractures using a combination of internal and external fixation is a simple technique that avoids the need for extensive soft tissue exposure while also promoting fracture stability and maintenance of bone length and rotation. This method can be incorporated into the armamentarium of orthopedic surgeons as an additional solution for addressing more challenging cases.

## 1. Introduction

Diaphyseal femoral fractures require submuscular plating, as fracture malunion and other surgical complications appear to be more prevalent. The fracture pattern, that is, whether the fracture is considered stable or unstable, is another important consideration. Stable diaphyseal fractures with transverse or short oblique patterns are relatively common injuries in the pediatric population, with a reported incidence of approximately 20 per 100,000 [1,2,3]. The accepted treatment for preschool children below 5 years of age is most commonly nonsurgical with spica casting, either immediately or after a few days of in-hospital skin traction [4]; for patients nearing the end of growth and beyond, adult-type surgical techniques of rigid intramedullary nailing are the common treatment. For patients aged 5–12 years, several treatment options may be considered without clear guidelines or strict treatment strategies; however, surgical treatment is generally mandatory.

The two most common fixation techniques are elastic intramedullary nailing (ESIN) and submuscular plating, with patient weight and fracture pattern being the two significant parameters influencing the surgeon’s decision. Heavy body weight (>50 kg) usually mandates a more rigid fixation; as such, non-obese patients are commonly treated with ESIN [5]. Conversely, unstable fractures, such as long, oblique, spiral, or comminuted, or fractures at the edges of the diaphysis, such as subtrochanteric and distal diaphyseal, are commonly treated with submuscular plating [6,7,8,9,10,11,12]. Each technique has disadvantages; residual angular malalignment and limb length discrepancy are attributed more to ESIN, whereas soft tissue damage and more extensile surgical exposure are attributed to submuscular plate fixation.

A third emerging option, more relevant to the upper part of this age group, involves the use of rigid intramedullary nailing, similar to adult intramedullary nailing. Limitations for the younger portion of this group include a small medullary canal diameter and potential harm to the trochanteric apophysis. Piriformis-fossa entry rigid nails were used in the past to avoid growth plate injuries but are less popular nowadays because of the risk of damaging the femoral head blood supply. Pediatric-adjusted lateral trochanteric entry points have become popular, with promising results regarding the potential risk of trochanteric growth arrest [13].

A fourth surgical option, the use of external fixation, is reserved for the deployed environment, damage control treatment, or severe soft tissue compromise and is usually a bridging solution before a definitive one [14,15]. Lastly, fewer documented surgical options were described by Anderson et al. in 2017 [16]. They described a combined method using both ESIN and an external fixator for unstable pediatric femoral fractures in two patients. However, only one of the two cases described was treated primarily with this method, whereas the second patient was treated with the combined method because of surgical complications after primary plate fixation. We present four additional cases of preadolescent children aged 9–12 years who sustained femoral diaphyseal fractures that were unstable or had severe soft tissue compromise and whose primary and definitive surgical treatment was combined with both ESIN and a uniplanar external fixator.

## 2. Patients and Methods

Publication of this paper was approved by the local IRB (institutional review board). Four patients aged 9–12 years who were operated on and followed postoperatively at our pediatric orthopedic unit between 2021 and 2023, were included in the study. All children were admitted to the hospital with isolated thigh trauma, underwent emergency care clearance, and were diagnosed with a diaphyseal femoral fracture with no additional concomitant injuries (Table 1).

The publication of the current paper was approved by the local institutional review board. Four patients, three boys and one girl aged 9–12 years, who were operated on and followed postoperatively at our pediatric orthopedic unit between the years 2021 and 2023, were included in the study. All children were initially admitted to the hospital emergency unit with isolated thigh trauma, underwent primary emergency care clearance, and were diagnosed with a diaphyseal femoral fracture with no additional concomitant injuries (Table 1).

### Surgical Technique

All patients were evaluated preoperatively and, owing to the characteristics of their fractures, were operated on using the combined technique of ESIN and a uniplanar external fixator as a primary surgical technique. All surgeries were performed by the same senior pediatric orthopedic surgical team. Following a satisfactory temporary closed reduction, two 3.5 mm elastic intramedullary nails (Titanium Elastic Nail (TEN), DePuy Synthes, Oberdorf, Switzerland) were inserted in an antegrade fashion from the distal metaphyseal femur, medially and laterally, through the fracture and proximally up to the trochanteric region.

**Figure 1:**
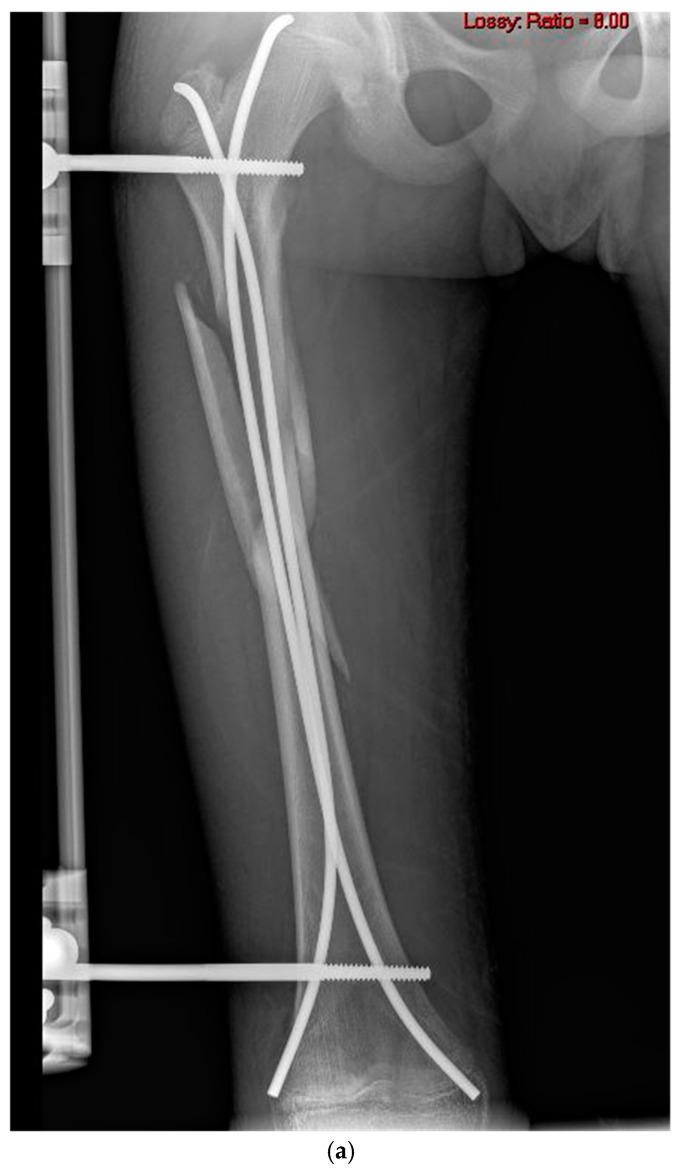

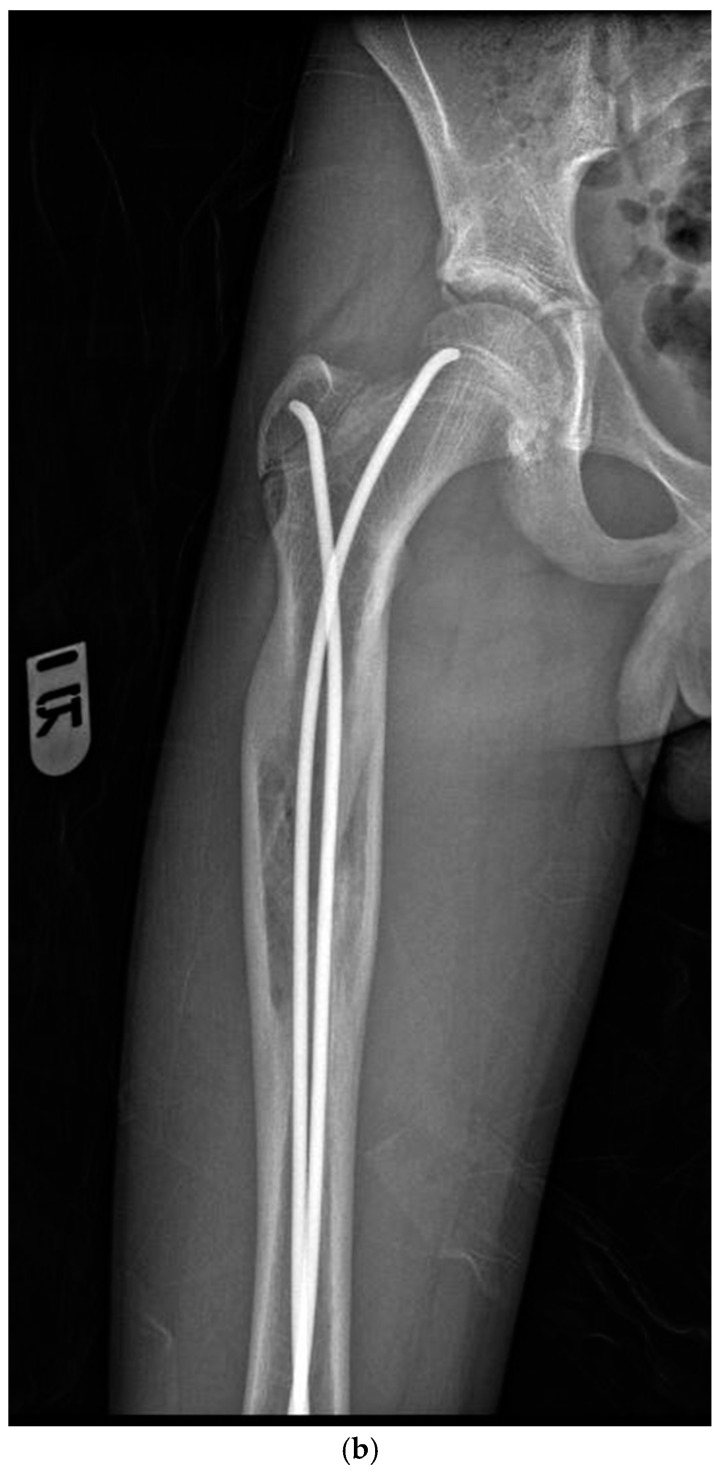

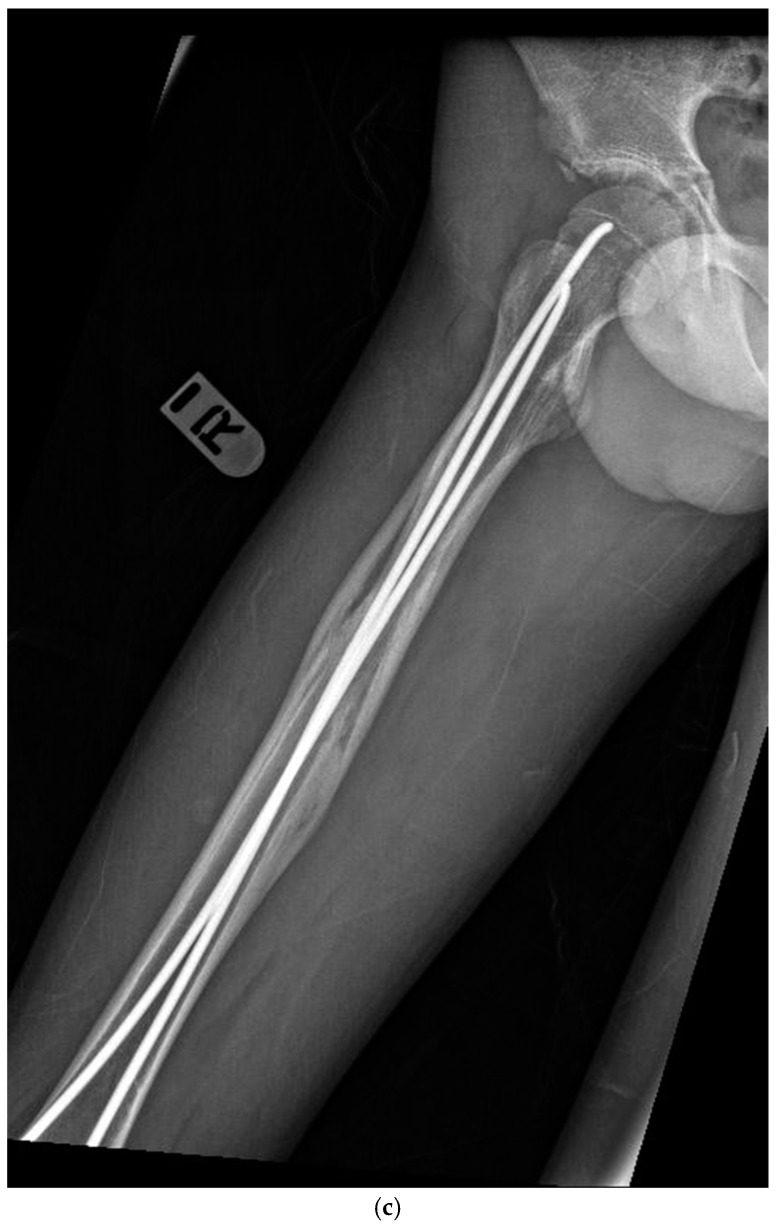
A 12-year-old male with a butterfly subtrochanteric fracture. (**a**) Immediate postoperative radiograph. (**b**,**c**) Twelve-week postoperative radiographs, anteroposterior and lateral views.

**Figure 2:**
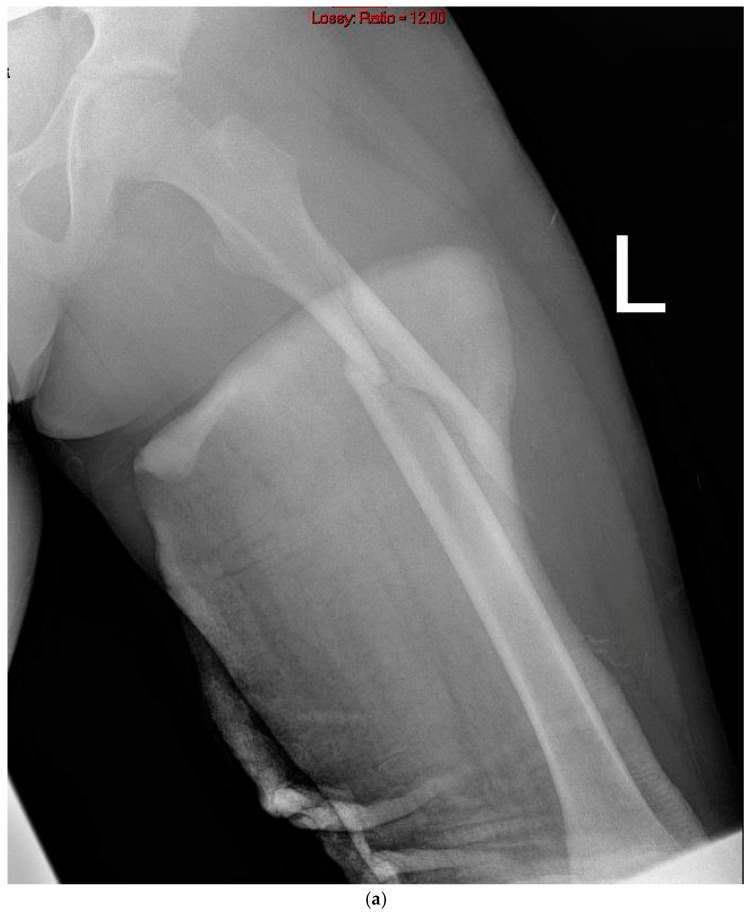

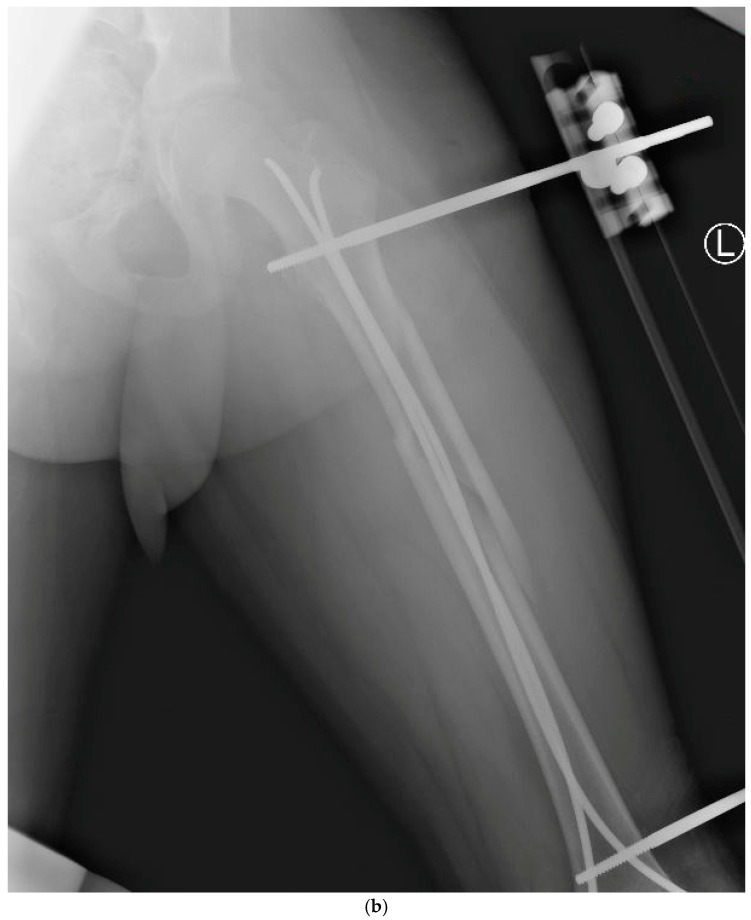

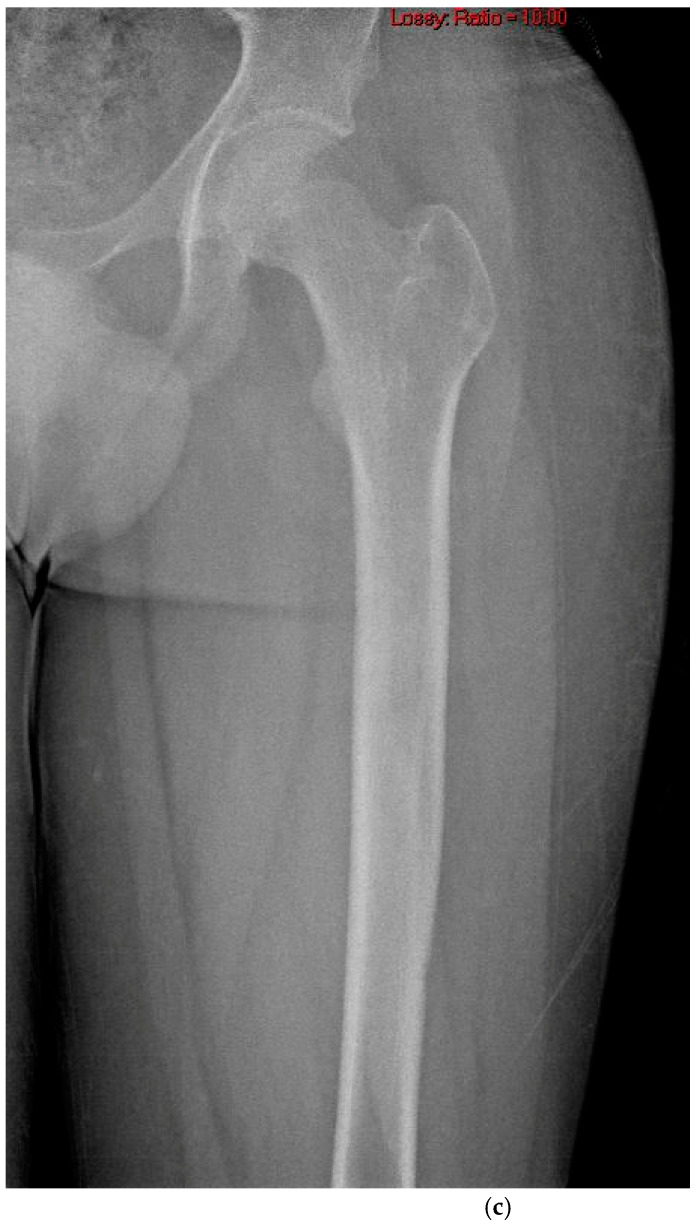
A 12.5-year-old with a subtrochanteric fracture with extension into the proximal femur. (**a**) Emergency room radiograph demonstrating a left subtrochanteric fracture with proximal extension. (**b**) Immediate postoperative radiograph with the uniplanar external fixator and two elastic intramedullary nails (ESINs). (**c**) After removal of the ESINs, with full fracture healing.

Owing to the fractures’ unstable patterns, two to four 6 mm hydroxyapatite-coated Schanz pins (OsteoTite pins, Orthofix, Lewisville, TX, USA) were implanted laterally, with one or two on each end of the fracture, and connected with a uniplanar external fixator (Orthofix Limb Reconstruction System (LRS), Orthofix, Lewisville, TX, USA) (Figure 1a and Figure 2a,b).

All patients received a non-weight-bearing regimen and were routinely followed up at our pediatric orthopedic outpatient clinic. Six to 12 weeks postoperatively, when satisfactory radiological healing was observed, the patient was admitted, and the external fixator was removed under general anesthesia (Figure 1b,c). Full weight-bearing with intense physiotherapy was initiated instantly. The patients were clinically and radiographically followed in a pediatric orthopedic clinic by the same team for a minimum of 12 months.

The four patients included in this study were evaluated preoperatively by the same pediatric orthopedic surgeons, and owing to the characteristics of their fractures and their age and weight, they were operated on using the combined technique of elastic intramedullary nails and a uniplanar external fixator as the primary surgical technique. All four surgeries were performed by the same senior pediatric orthopedic surgical team. Following a satisfactory temporary closed reduction under fluoroscopy, two 3.5 mm elastic intramedullary nails (Titanium Elastic Nail (TEN), DePuy Synthes, USA) were inserted in a retrograde fashion from the distal metaphyseal femur medially and laterally, using minimal incisions, through the fracture site, and proximally up to the trochanteric region.

Owing to the fractures’ unstable patterns, which were demonstrated both before and after operation, further torsional stability and length control were needed; therefore, two or four 6 mm hydroxyapatite-coated Schanz pins (OsteoTite pins, Orthofix, USA) were implanted from the lateral side of the femur through minimal incisions. Each Schanz pin was carefully inserted with accurate perpendicular bicortical fixation, with one or two pins on each end of the fracture. The pins were inserted at a posterior position to the elastic nails to prevent any contact between the external device and the internal fixation. Finally, all the Schanz pins were connected together with a uniplanar external fixator (Orthofix Limb Reconstruction System (LRS), Orthofix, USA) (Figure 1a and Figure 2a,b).

Postoperatively, all patients received a non-weight-bearing regimen with meticulous physiotherapy and were routinely followed up at our pediatric orthopedic outpatient clinic. Six to twelve weeks postoperatively, when satisfactory radiological healing was observed, the patient was admitted to the hospital and the external fixator was removed under general anesthesia (Figure 1b,c). Full weightbearing with further intense physiotherapy was initiated instantly. The patients were clinically and radiographically followed in a pediatric orthopedic clinic by the same team for a minimum of 12 months.

## 3. Results

None of the four patients in our case series had any major surgical or injury-related complications, including refracture, deep infection, vascular injury, neurologic deficits, or chronic pain. Two patients experienced knee stiffness prior to the removal of the external fixator, but following the physiotherapy regimen, both gained full range of motion within a few months. One patient was diagnosed with a pin-tract infection a few days before the six-week postoperative follow-up. Foul odor with cloudy discharge from the pin wounds warranted the admittance of the patient to the orthopedic department, and the external fixator was removed. The infection resolved following a week of intravenous antibiotics followed by oral antibiotics. Two patients who were symptomatic due to prominent ESINs proximal to the knee joints at the distal thigh, had them removed under general anesthesia 14 months postoperatively (Figure 1c). All patients regained a fully symmetrical range of motion of the hip and knee joints. No patient had a clinically significant limb length discrepancy or angular deformity.

## 4. Discussion

Treating femoral fractures in the pediatric population can be challenging. Due to the high bone remodeling potential at preschool age, nonoperative solutions are acceptable or even recommended, while at older ages, nonunion, residual axial deformity, rotational deformity, or limb-length discrepancy are worrisome complications that often mandate surgery [17,18].

The choice of technical solution varies according to the patient’s age, body weight, and fracture pattern. However, no significant overall advantage has been shown regarding the chosen method [19], and each method has benefits and disadvantages. Narayanan et al. [20] demonstrated that femoral fracture fixation with intramedullary elastic nails alone, although technically relatively simple with minimal soft tissue and bone exposure, has relatively high complication rates, with up to 50% of prominent symptomatic nail edges, and a high rate of malunion, with approximately 12% of patients requiring reoperation prior to union. For comminuted fractures, which are unstable, the group recommends stricter post-operative follow-up and further immobilization. Submuscular plating for unstable pediatric femoral fractures seems to have fewer complications than ESIN [21] but requires a longer operative time for both the initial fixation and the removal of the plate [9]. In fractures where submuscular plates are implanted, a second surgery is recommended, especially if the implant is relatively distal. Although the removal of the plate can be time-consuming and technically more challenging with relatively extensile soft tissue exposure, the retention of submuscular plates may result in angular deformities, leg-length discrepancies, or prominent hardware [22].

In our case series, we described an alternative for the primary treatment of the more complex femoral diaphyseal fractures, using a combined surgical technique of both elastic intramedullary nails and a uniplanar external fixator, in a fashion similar to that previously described in one of two cases by Anderson et al. [16]. The advantage of using an external fixator for femoral fracture reduction has been previously described by our group [23]; however, in the current technique, the fixator, beyond being a temporary assisting tool for reduction and rotational control, is also part of the final fracture fixation. In our opinion, in correctly selected cases of preadolescent complex unstable or comminuted diaphyseal femoral fractures, this technique is appropriate and allows for minimal surgical exposure, minimal blood loss, no soft tissue stripping, and relatively affordable and more importantly available hardware. In a biomechanical study by Wilton et al. [24], the augmentation of elastic nails with an external fixator demonstrated excellent stability and rotational control, potentially eliminating some of the main disadvantages of ESIN alone [24]. Early and technically simple removal of the external fixator permits good rehabilitation and decreases the risk of infection while maintaining the elastic nails facilitates additional stability until full healing is observed.

However, we acknowledge that the disadvantages of this technique should be considered. The need for at least one additional general anesthesia for external fixator removal and the risk of pin tract infection are not negligible considerations. Careful insertion of the implants without any direct contact is important to reduce the risk of intramedullary infection. Nonetheless, on the basis of our experience, we believe that this technique should be part of the pediatric orthopedic trauma surgeon armamentarium for treating diaphyseal femoral fractures, as it provides more versatility in treatment options, especially in more challenging cases of pediatric preadolescent femoral fractures.

## 5. Conclusions

Complex unstable diaphyseal fractures in the preadolescent population poses a technical challenge for the treating surgeon. At this age, there are several surgical solutions, each with benefits and weaknesses. We hope that the combined method described in our study of fracture fixation using an external fixator with elastic intramedullary nails adds a substantially useful tool that expands the number of available surgical solutions and offers yet another appropriate treatment option.

## Figures and Tables

**Table 1:** Characteristics of pediatric patients with diaphyseal femoral fractures treated with ESINs and an external fixator.

No.	Age (Years)	Gender	Side	Fracture Pattern	Cause	Surgical Technique	Removal of Ex-Fix (Months)	Removal of ESINs (Months)	Complications
1	9.8	Male	R	Mid-diaphyseal long spiral	Direct hit	Retrograde ESINs and uniplanar Ex-Fix	1.5	7	Pin-tract infection
2	10.3	Female	L	Distal diaphyseal long spiral	Fall	Retrograde ESINs and uniplanar Ex-Fix	3	17	Irritable ESIN LLD 0.5cm
3	12.5	Male	L	Comminuted subtrochanteric	Fall	Retrograde ESINs and uniplanar Ex-Fix	2	14	Irritable ESIN
4	12.5	Male	R	Butterfly, subtrochanteric	Fall from horse	Retrograde ESINs and uniplanar Ex-Fix	1.8	n/a	none

## Data Availability

The raw data supporting the conclusions of this article will be made available by the authors on request.
